# Combination of serum AIM2 and caspase-5 as a predictor of unfavorable prognosis in children with lobar pneumonia: a prospective cohort study

**DOI:** 10.3389/fped.2026.1907114

**Published:** 2026-10-14

**Authors:** Linlin Liu, Xiaoxue Qi, Liping Zhang, Jianfang Jiang, Wenshan Lyu, Yuxiao Hu, Peiqing Huo

**Affiliations:** 1 Respiratory Department II, Hebei Children's Hospital, Shijiazhuang, Hebei, China; 2 Respiratory Department III, Hebei Children's Hospital, Shijiazhuang, Hebei, China; 3 Department of Pediatrics Ward 1, Shexian Hospital, Handan, China

**Keywords:** lobar pneumonia, AIM2, caspase-5, prognosis, pediatric

## Abstract

**Objective:**

To validate whether serum levels of absent in melanoma 2 (AIM2) and caspase-5 can serve as early combined biomarkers of unfavorable prognosis in children with lobar pneumonia.

**Methods:**

From February 2022 to June 2025, 475 children aged 1–14 years with first-episode lobar pneumonia were prospectively enrolled at Hebei Children's Hospital. Patients were divided into a favorable-prognosis group (*n* = 395) and an unfavorable-prognosis group (*n* = 80) based on treatment outcomes assessed during outpatient follow-up visits conducted 4–6 weeks after discharge. Fasting venous blood was drawn within 24 h of admission, and levels of AIM2 and caspase-5 were measured by enzyme-linked immunosorbent assay (ELISA). Co-infection, symptom duration, antibiotic duration, hospital stay, C-reactive protein (CRP), procalcitonin (PCT), and lactate dehydrogenase (LDH) were recorded. Prognostic associations were examined using Pearson correlation, multivariable logistic regression, and receiver operating characteristic (ROC) analysis. Multicollinearity was assessed; a tolerance > 0.1 and a variance inflation factor (VIF) < 10 were considered acceptable.

**Results:**

Serum AIM2 and caspase-5 levels were significantly higher in the unfavorable-prognosis group than in the favorable-prognosis group (both *P* < 0.001), and the two markers were positively correlated (*r* = 0.241, *P* < 0.001). Multivariable analysis identified co-infection and elevated levels of CRP, PCT, AIM2, and caspase-5 as independent risk factors for unfavorable prognosis. The VIF ranged from 1.047 to 1.131, indicating no multicollinearity. The combination of AIM2 and caspase-5 yielded an AUC of 0.839 (95% CI 0.798–0.879) for predicting unfavorable prognosis, with a sensitivity of 75.70% and a specificity of 78.75%, which was significantly superior to either biomarker alone (DeLong test: Z = −4.248, *P* < 0.001; Z = −2.998, *P* = 0.003). Nested model analysis demonstrated that adding AIM2 and caspase-5 to conventional variables (co-infection, CRP, PCT, and LDH) improved the AUC from 0.855 to 0.893 (DeLong test *P* = 0.047), indicating significant incremental predictive value.

**Conclusion:**

Elevated serum AIM2 and caspase-5 are independent risk factors for unfavorable prognosis in children with lobar pneumonia. Their combination shows favorable predictive performance and may help identify high-risk children early.

## 1 Introduction

Lobar pneumonia is an acute respiratory infection frequently encountered in children, pathologically characterized by consolidative changes involving one or more pulmonary lobes or segments (1, 2). The incidence of this disease in the pediatric population has increased in recent years (3). Although most patients respond favorably to timely antibiotic therapy, some cases progress rapidly and, without prompt intervention, may develop severe complications including pleural effusion, lung abscess, and even respiratory failure, thereby posing a substantial threat to life and health (4–6). In the present study, poor prognosis was defined as the absence of symptomatic and radiographic improvement at 4 to 6 weeks post-discharge, to distinguish this outcome from acute-phase complications such as empyema, ICU admission, or mechanical ventilation. Because the prognosis of lobar pneumonia is closely associated with the quality of life and long-term health status of pediatric patients (7, 8), achieving early risk assessment and predicting disease outcomes have become critical issues in current clinical research, motivating the exploration of predictive biomarkers to facilitate clinical decision-making and optimize therapeutic strategies.

Absent in melanoma 2 (AIM2), a critical inflammasome sensor, activates caspase-1 through the canonical pathway, thereby promoting the maturation and secretion of the inflammatory cytokines interleukin (IL)-1β and IL-18, and participates in cytosolic DNA recognition and the regulation of inflammatory responses (9). Caspase-5 is a cysteine protease that serves as an effector protease of the noncanonical inflammasome pathway; its caspase recruitment domain (CARD) directly recognizes cytosolic lipopolysaccharide (LPS), whereas caspase-5 itself mediates pyroptosis via cleavage of gasdermin D (GSDMD), thereby facilitating the release of proinflammatory cytokines (10, 11). These two molecules are components of distinct inflammasome activation pathways and may be simultaneously activated in severe pulmonary infection. As representative serum biomarkers of their respective pathways, they collectively reflect the overall activation status of inflammasomes.

The predominant pathogens responsible for lobar pneumonia in children include Streptococcus pneumoniae and Mycoplasma pneumoniae. Previous studies have shown that after S. pneumoniae is internalized by macrophages, its DNA is released into the cytoplasm in a pneumolysin-mediated manner, where it is recognized by AIM2, thereby activating the caspase-1-dependent inflammasome and promoting IL-1β maturation. Murine model studies have further demonstrated that the AIM2 inflammasome participates in intrapulmonary bacterial clearance and regulates pulmonary inflammation (12). Current cellular and animal model studies have indicated that M. pneumoniae membrane lipoproteins and the CARDS toxin can activate the NLRP3 inflammasome, promoting inflammatory cytokine release and macrophage pyroptosis, thereby exacerbating lung tissue injury. This inflammatory amplification mechanism may also be involved in the progression of refractory Mycoplasma pneumoniae pneumonia (RMPP); however, current evidence is derived primarily from models of uncomplicated MPP (13). Both the canonical caspase-1-dependent inflammasome pathway and the noncanonical caspase-4/5/11 pathway can induce pyroptosis through GSDMD cleavage, releasing proinflammatory cytokines such as IL-1β and IL-18. In acute lung injury, pyroptosis damages pulmonary vascular endothelial and epithelial cells, aggravating alveolar-capillary barrier disruption and leading to interstitial edema and inflammatory exudation (14). Massive IL-1β release driven by the former pathway may promote pleural effusion formation, whereas pyroptosis mediated by the latter pathway may result in persistent non-resolution of pulmonary consolidation, increasing the risk of lung abscess and respiratory failure. Therefore, serum AIM2 and caspase-5 levels may reflect the extent of activation of these two pathways, respectively, and together predict the severity of lung tissue injury and the risk of complications.

Recently, Hu et al. found that serum levels of AIM2 were associated with complicated community-acquired pneumonia (CAP) in 305 pediatric patients with CAP (15). However, their study enrolled a broad CAP cohort rather than the lobar pneumonia subtype, assessed AIM2 alone, and evaluated only acute in-hospital complications rather than long-term radiographic recovery. Therefore, the value of AIM2 combined with caspase-5 in predicting long-term prognosis in children with lobar pneumonia remains unclear. Building upon this prior work, the present study specifically focuses on the lobar pneumonia subtype, adopts a dual-biomarker approach using AIM2 and caspase-5, and defines long-term prognosis as radiographic recovery 4–6 weeks after discharge, thereby filling this gap. This study aims to investigate the association of serum AIM2 and caspase-5 levels with disease severity in children with lobar pneumonia, as well as their predictive value for prognosis, to provide novel biomarker evidence for the early clinical identification of high-risk pediatric patients and for facilitating risk stratification, and to lay the groundwork for subsequent intervention studies aimed at improving long-term prognosis and quality of life.

## 2 Study population and methods

### 2.1 Sample size calculation

A previous study (8) reported an unfavorable prognosis rate of approximately 16% in children with lobar pneumonia. To limit the margin of error to ±5% (i.e., a 95% CI width of 10%), we set *α* = 0.05 (two-sided) and used PASS 15.0. The calculation indicated that at least 225 participants would be required to reliably estimate the event rate with the predetermined precision, thereby ensuring such precision and scientific rigor of the study conclusions.

### 2.2 Inclusion and exclusion criteria

Inclusion criteria: (1) Diagnosis of pediatric CAP according to the diagnostic criteria outlined in the Guidelines for the Management of Community-acquired Pneumonia in Children, with radiological manifestations of lobar pneumonia: (a) acute onset of fever, cough, and tachypnea, with auscultatory findings of moist rales and/or signs of pulmonary consolidation (bronchial breathing sounds, dullness to percussion); (b) chest radiography or computed tomography (CT) demonstrating homogeneous dense consolidation of a pulmonary lobe or segment, with or without air bronchogram, pleural effusion, or atelectasis; (c) without radiological evidence of lobular (patchy, punctate) infiltration, interstitial changes, or mixed infiltrative pattern with predominant interstitial involvement; (2) Age 1–14 years, with a first-time diagnosis of lobar pneumonia and no other prior targeted treatment; (3) Complete clinical documentation available, including detailed medical history, physical examination findings, laboratory data, imaging reports, and information on treatment course and prognostic follow-up.

Exclusion criteria: (1) Congenital heart disease; (2) Chronic pulmonary disease, immunosuppression, or use of antibiotics or corticosteroids prior to admission; (3) Hepatic, renal, or other major organ dysfunction; (4) Autoimmune diseases, hematological disorders, or other diseases affecting additional organ systems; (5) Malignancy; (6) Mental disorders or cognitive impairment; (7) Concurrent acute infections at other sites (e.g., purulent meningitis, sepsis, complicated urinary tract infection, active tuberculosis); (8) Receipt of live-attenuated vaccines within the past month or recent participation in other clinical trials of drugs or biomarkers; (9) Severe malnutrition or severe anemia at the time of admission.

### 2.3 Patients

This prospective observational study enrolled children with lobar pneumonia treated at the Hebei Children's Hospital from February 2022 to June 2025. Of 518 patients initially enrolled, 475 were included after application of the exclusion criteria (Figure 1). Of the 43 excluded patients, 8 did not meet the diagnostic criteria for lobar pneumonia, 3 had congenital heart disease, 4 had chronic lung disease, 5 had immunosuppression, 9 had received antibiotics or corticosteroids prior to admission, 2 had dysfunction of the liver, kidneys, or other vital organs, 1 had malignancy, 2 had mental disorders or cognitive impairment, 3 had autoimmune diseases, hematological disorders, or other systemic diseases, 4 had acute infections at other sites, and 2 had received live-attenuated vaccines within the previous month.

**Figure 1:**
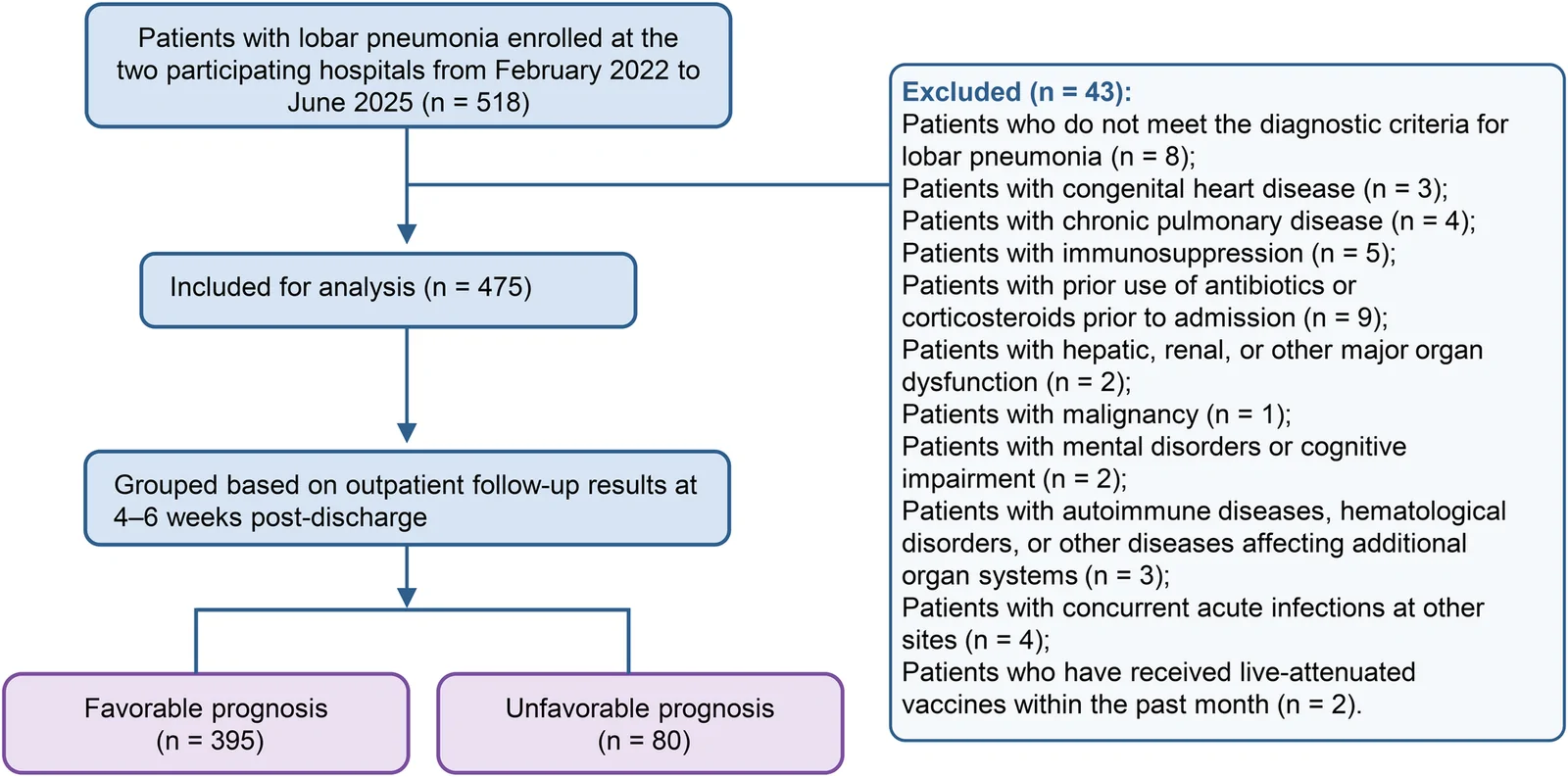
Flow diagram of patient enrollment and group allocation.

Based on treatment outcomes at outpatient follow-up 4–6 weeks after discharge, patients were classified as cured (complete resolution of symptoms and radiographic recovery), improved (partial resolution of symptoms and lesion absorption), or ineffective (no significant change) (8). Patients who were cured or improved were classified into the favorable-prognosis group (*n* = 395), whereas those with ineffective treatment were classified into the unfavorable-prognosis group (*n* = 80). All 475 included patients completed outpatient follow-up at 4–6 weeks after discharge, with complete laboratory data and no loss to follow-up or missing values.

This study was conducted in accordance with the Declaration of Helsinki and approved by the Medical Ethics Committees of the Hebei Children's Hospital. Written informed consent was obtained from the guardians of all patients. All patient data were anonymized to protect patient privacy.

### 2.4 Clinical data collection

Clinical data were retrieved from the hospital medical records database. Variables included sex, age, disease course, body mass index (BMI), infected pulmonary lobe, co-infection status (defined as the simultaneous detection of two or more pathogens, such as bacteria combined with Mycoplasma or viruses), clinical manifestations, and laboratory findings. Clinical manifestations included the duration of fever, cough, pulmonary rales, antibiotic therapy, and hospital stay. Laboratory findings measured at admission included white blood cell (WBC) count, hemoglobin (Hb), platelet count (PLT), C-reactive protein (CRP), procalcitonin (PCT), lactate dehydrogenase (LDH), alanine aminotransferase (ALT), and aspartate aminotransferase (AST).

### 2.5 Measurement of serum AIM2 and caspase-5

Fasting venous blood samples (5 mL) were collected within 24 h of admission and prior to antibiotic administration. Samples were left at room temperature for 2 h or overnight at 4 °C, then centrifuged at 4 °C (2,500–3,500 × g for 20 min). The resulting serum was aliquoted and stored at –80 °C. After collection, samples were visually inspected to exclude those with obvious hemolysis or lipemia. Serum quality was reassessed after centrifugation, and samples failing quality control were excluded. Serum levels of AIM2 and caspase-5 were measured by enzyme-linked immunosorbent assay (ELISA). ELISA kits (Wuhan Beiyinlai Biotechnology; AIM2, cat. no. HM12011; caspase-5, cat. no. HM11810) were used according to the manufacturer's instructions. The detection ranges for both AIM2 and caspase-5 were 0.125–10 ng/mL, with sensitivities of ≤0.025 ng/mL. The reaction time was 1 h, and the sample volume was 40 μL. According to the manufacturer's validation data, both kits exhibited good specificity. The AIM2 kit specifically detects human AIM2 with no significant cross-reactivity with related PYHIN family proteins, and the caspase-5 kit specifically detects human caspase-5 with no significant cross-reactivity with related caspase-4/11 proteins. Intra-assay and inter-assay coefficients of variation were both <10%, meeting the precision standards for ELISA. Samples were stored for no longer than 6 months. Samples were tested in batches according to date of collection. Each batch included standards in duplicate and quality control samples to monitor inter-batch consistency. All samples were subjected to a single freeze-thaw cycle (thawed once immediately prior to analysis) to avoid repeated freeze-thawing.

Prior to testing, kits were equilibrated to room temperature. A sandwich ELISA was performed by sequentially adding standards, serum samples, biotin-labeled antibody, and enzyme-labeled reagents. After color development, absorbance [optical density (OD)] was measured at 450 nm, and concentrations were calculated from the standard curve. All samples were tested in duplicate. Samples with a coefficient of variation between duplicates exceeding 15% were reanalyzed. All measured values fell within the quantitative range of the kits (0.125–10 ng/mL), and no values below the lower limit of quantification required special handling. Testing was performed by laboratory personnel blinded to group allocation. Samples were identified only by random numbers, and blinding was maintained until database lock.

### 2.6 Follow-up and outcome assessment

All children met the criteria for clinical stability at discharge: absence of fever for ≥48 h, respiratory stability, tolerance of oral intake, and peripheral oxygen saturation (SpO_2_) ≥ 95%. At 4–6 weeks after discharge, the children underwent outpatient follow-up chest radiography. Lesion resolution was defined as the absence of both residual infiltrates and fibrotic foci in the originally consolidated area. The imaging results were independently interpreted by two senior radiologists who were blinded to serum AIM2 and caspase-5 assay results, as well as clinical group assignments. Discrepancies between the two radiologists were resolved by a third senior radiologist.

### 2.7 Statistical analysis

Statistical analyses were performed using SPSS 27.0, and figures were prepared using GraphPad Prism 10.0. The Kolmogorov–Smirnov test was used to assess the normality of continuous variables. Normally distributed continuous variables were expressed as mean ± standard deviation (SD), and between-group comparisons were performed using the t-test. Non-normally distributed continuous variables were presented as median with interquartile range [M (Q1, Q3)], and between-group comparisons were conducted using the Mann–Whitney *U* test. Categorical variables were expressed as count and percentage (%), and between-group comparisons were performed using the *χ*^2^ test. For correlation analysis, Pearson correlation was used for normally distributed variables and Spearman correlation for non-normally distributed variables. Logistic regression analysis was performed to identify risk factors for prognosis in children with lobar pneumonia. Multicollinearity among the selected variables was assessed; tolerance > 0.1 and variance inflation factor (VIF) < 10 indicated the absence of multicollinearity. ROC curves were constructed to evaluate the predictive value of serum AIM2 and caspase-5 levels for prognosis in children with lobar pneumonia. To assess the incremental predictive value of AIM2 and caspase-5 beyond traditional inflammatory markers, two nested logistic models were constructed: Model 1 (base model) included co-infection, CRP, PCT, and LDH; Model 2 (extended model) added AIM2 and caspase-5 to Model 1. The DeLong test was used to compare the AUCs of the two models. Statistical significance was set at *P* < 0.05.

## 3 Result

### 3.1 Comparison of clinical characteristics between the two groups

All 475 patients completed outpatient follow-up 4–6 weeks after discharge; the follow-up time was 4.0–6.0 weeks (median, 5.0 weeks). The favorable-prognosis group comprised 395 patients (83.16%), of whom 347 were cured and 48 improved; the unfavorable-prognosis group comprised 80 patients (16.84%), all classified as ineffective.

Compared with the favorable-prognosis group, the unfavorable-prognosis group had a significantly higher proportion of co-infections (simultaneous detection of two or more pathogens, such as bacterial infection combined with Mycoplasma or viral infection) (*P* < 0.001). The durations of fever (*P* = 0.014), cough (*P* = 0.013), pulmonary rales (*P* < 0.001), antibiotic use (*P* < 0.001), and hospital stay (*P* < 0.001) were significantly prolonged. Laboratory findings showed that levels of CRP, PCT, and LDH were significantly higher in the unfavorable-prognosis group than in the favorable-prognosis group (all *P* < 0.001), with no significant differences in other parameters (*P* > 0.05) (Table 1).

**Table 1:** Comparison of clinical characteristics between the two groups.

Variable	Unfavorable-prognosis group (*n* = 80)	Favorable-prognosis group (*n* = 395)	Test statistic	*P* values
Sex, n (%)			*χ*^2^ = 0.069	0.792
Male	42 (52.50)	201 (50.89)		
Female	38 (47.50)	194 (49.11)		
Age [median (Q1, Q3), years]	7.00 (6.00,8.00)	7.00 (6.00,9.00)	Z = -0.876	0.381
Height [median (Q1, Q3), cm]	114.50 (112.00,118.00)	114.00 (112.00,117.00)	Z = 0.513	0.608
Weight (mean ± SD, kg)	25.12 ± 4.91	25.14 ± 5.07	t = -0.032	0.974
Co-infection, n (%)			χ^2^ = 43.542	<0.001
Yes	63 (78.75)	152 (38.48)		
No	17 (21.25)	243 (61.52)		
Clinical manifestations				
Infected pulmonary lobe, n (%)			χ^2^ = 0.531	0.767
Left	38 (47.50)	175 (44.30)		
Right	35 (43.75)	190 (48.10)		
Bilateral	7 (8.75)	30 (7.59)		
Duration of fever [median (Q1, Q3), days]	3.00 (2.00,4.00)	3.00 (2.00,4.00)	Z = -2.451	0.014
Duration of cough [median (Q1, Q3), days]	8.00 (7.00,8.75)	7.00 (7.00,8.00)	Z = -2.482	0.013
Duration of pulmonary rales [median (Q1, Q3), days]	8.00 (7.00,9.00)	7.00 (6.00,8.00)	Z = -5.359	<0.001
Duration of antibiotic use [median (Q1, Q3), days]	11.00 (10.00,12.00)	8.00 (7.00,9.00)	Z = -12.332	<0.001
Length of hospital stay [median (Q1, Q3), days]	13.00 (12.00,14.00)	10.00 (9.00,11.00)	Z = -12.261	<0.001
Laboratory findings				
WBC (mean ± SD, 10^9^/L)	13.04 ± 3.93	13.18 ± 3.89	t = -0.294	0.769
Hb (mean ± SD, g/L)	128.63 ± 10.71	126.81 ± 9.88	t = 1.477	0.140
PLT (mean ± SD, 10^9^/L)	386.85 ± 76.53	384.77 ± 78.65	t = 0.216	0.829
CRP (mean ± SD, mg/L)	25.61 ± 2.38	23.05 ± 2.43	t = 8.621	<0.001
PCT [median (Q1, Q3), ng/mL]	5.88 (5.12,6.55)	3.94 (3.03,4.67)	Z = -10.775	<0.001
LDH (mean ± SD, U/L)	278.02 ± 22.18	266.01 ± 23.46	t = 4.213	<0.001
ALT [median (Q1, Q3), U/L]	36.00 (32.00,42.75)	37.00 (32.00,42.00)	Z = -0.586	0.558
AST (mean ± SD, U/L)	24.58 ± 6.44	23.89 ± 6.68	t = 0.844	0.399

Normally distributed continuous data are expressed as mean ± standard deviation (SD) and analyzed using the independent samples t-test; non-normally distributed continuous data are expressed as median (first quartile [Q1], third quartile [Q3]) and analyzed using the Mann–Whitney U test (Z values reported); categorical data are analyzed using the χ^2^ test.

WBC, white blood cell; Hb, hemoglobin; PLT, platelet count; CRP, C-reactive protein; PCT, procalcitonin; LDH, lactate dehydrogenase; ALT, alanine aminotransferase; AST, aspartate aminotransferase.

### 3.2 Comparison of serum AIM2 and caspase-5 levels between the two groups

Serum AIM2 and caspase-5 levels were significantly higher in the unfavorable-prognosis group than in the favorable-prognosis group (both *P* < 0.001). The Kolmogorov–Smirnov test showed that both AIM2 (*P* = 0.084) and caspase-5 (*P* = 0.200) were normally distributed; therefore, Pearson correlation analysis was performed. The analysis revealed a positive correlation between serum AIM2 and caspase-5 levels in children with lobar pneumonia (*r* = 0.241, *P* < 0.001) (Figure 2).

**Figure 2:**
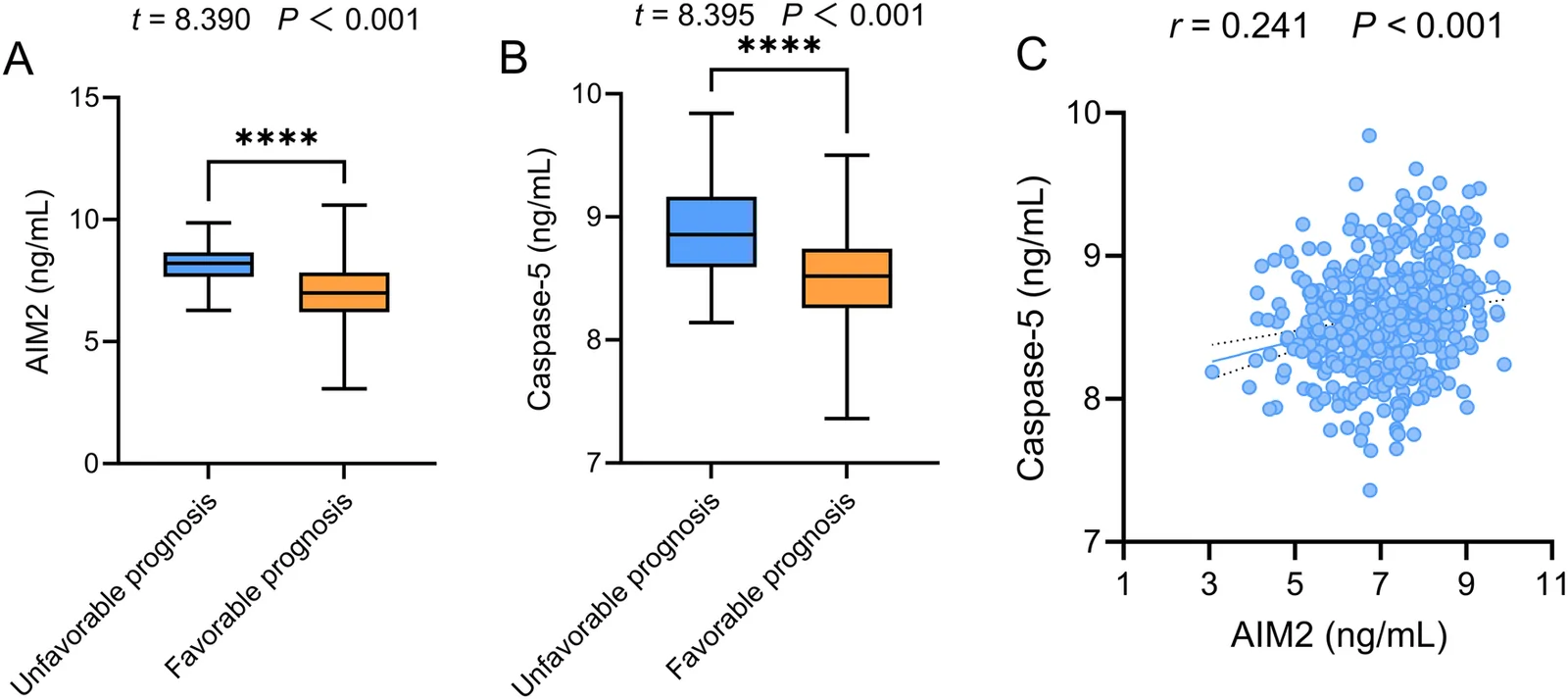
Comparison of serum AIM2 and caspase-5 levels between the two groups and their correlation. **(A)** Comparison of serum AIM2 levels between the unfavorable-prognosis group (*n* = 80) and the favorable-prognosis group (*n* = 395); **(B)** comparison of serum caspase-5 levels between the unfavorable-prognosis group and the favorable-prognosis group; **(C)** correlation analysis between serum AIM2 and caspase-5 levels in children with lobar pneumonia. ****P < 0.001.

### 3.3 Multivariable logistic regression analysis of factors affecting unfavorable prognosis in children with lobar pneumonia

Multivariable logistic regression analysis using the enter method was performed with the prognosis of children with lobar pneumonia as the dependent variable (unfavorable prognosis = 1, favorable prognosis = 0) and co-infection status (yes = 1, no = 0), CRP, PCT, LDH, and serum AIM2 and caspase-5 levels as independent variables. The results showed that co-infection and elevated CRP, elevated PCT, and elevated serum AIM2 and caspase-5 levels were risk factors for unfavorable prognosis in children with lobar pneumonia, whereas LDH was not significantly associated with unfavorable prognosis (*P* = 0.174). Multicollinearity diagnostics showed that tolerance values for the six included variables ranged from 0.884 to 0.955, and VIFs ranged from 1.047 to 1.131, indicating no multicollinearity (Table 2; Figure 3).

**Table 2:** Multivariable logistic regression analysis of factors affecting unfavorable prognosis in children with lobar pneumonia.

Variable	*β*	*S.E*	*Wald*	*P*	*OR*	95% CI	Tolerance	VIF
Co-infection status	1.552	0.459	11.418	0.001	4.723	1.919-11.622	0.940	1.064
CRP	0.457	0.103	19.579	<0.001	1.580	1.290-1.935	0.937	1.067
PCT	1.269	0.202	39.418	<0.001	3.558	2.394-5.288	0.884	1.131
LDH	0.012	0.009	1.849	0.174	1.013	0.995-1.031	0.955	1.047
AIM2	0.846	0.220	14.726	<0.001	2.329	1.512-3.587	0.934	1.071
Caspase-5	0.959	0.150	41.101	<0.001	2.610	1.947-3.500	0.938	1.066

CRP, C-reactive protein; PCT, procalcitonin; LDH, lactate dehydrogenase; AIM2, Absent in melanoma 2.

**Figure 3:**
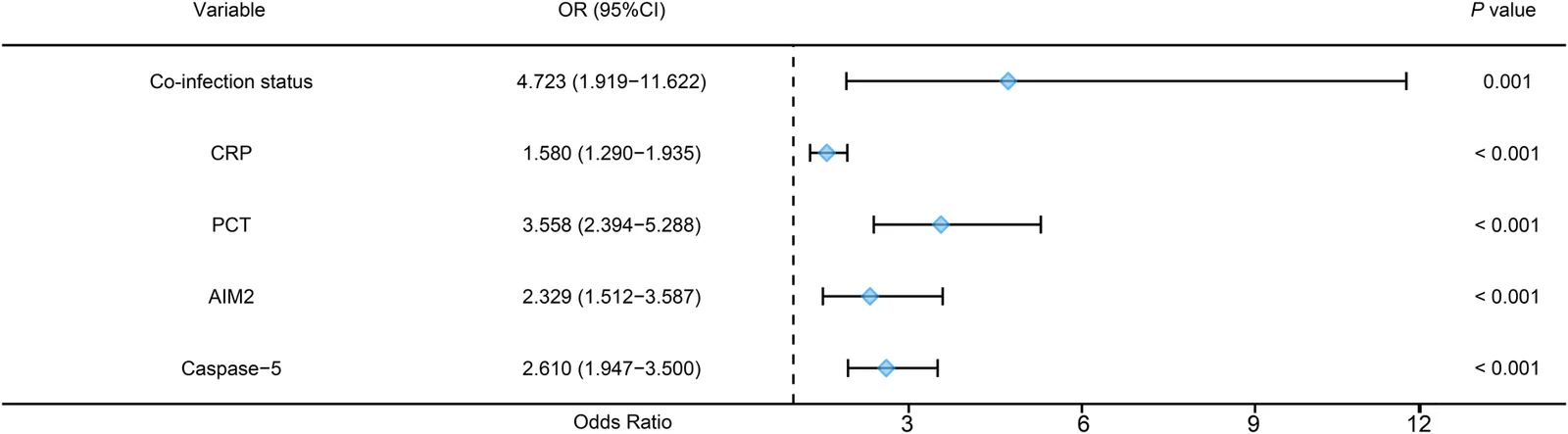
Forest plot of multivariable logistic regression analysis of factors associated with unfavorable prognosis in children with lobar pneumonia. Only variables with statistical significance (*P* < 0.05) are displayed in the figure. LDH was not statistically significant (OR = 1.013, 95% CI 0.995–1.031, *P* = 0.174) and was not included in the figure.

### 3.4 Predictive value of serum AIM2 and caspase-5 for unfavorable prognosis in children with lobar pneumonia

ROC curve analysis showed that the combination of serum AIM2 and caspase-5 yielded an AUC of 0.839 for predicting unfavorable prognosis in children with lobar pneumonia. DeLong test indicated that the AUC of the combination was significantly higher than that of AIM2 alone (Z = −4.248, *P* < 0.001) and that of caspase-5 alone (Z = −2.998, *P* = 0.003) (Table 3; Figure 4).

**Table 3:** Predictive value of serum AIM2 and caspase-5 for unfavorable prognosis in children with lobar pneumonia.

*Variable*	Cut-off	Sensitivity (%)	Specificity (%)	AUC	95% CI
*AIM2*	7.615 ng/mL	70.13	76.25	0.797	0.751-0.842
*Caspase-5*	8.705 ng/mL	71.14	62.50	0.756	0.698-0.815
*AIM2 + Caspase-5*	-	75.70	78.75	0.839	0.798-0.879

AIM2, Absent in melanoma 2; AUC, area under the curve; CI, confidence interval; ROC, receiver operating characteristic.

**Figure 4:**
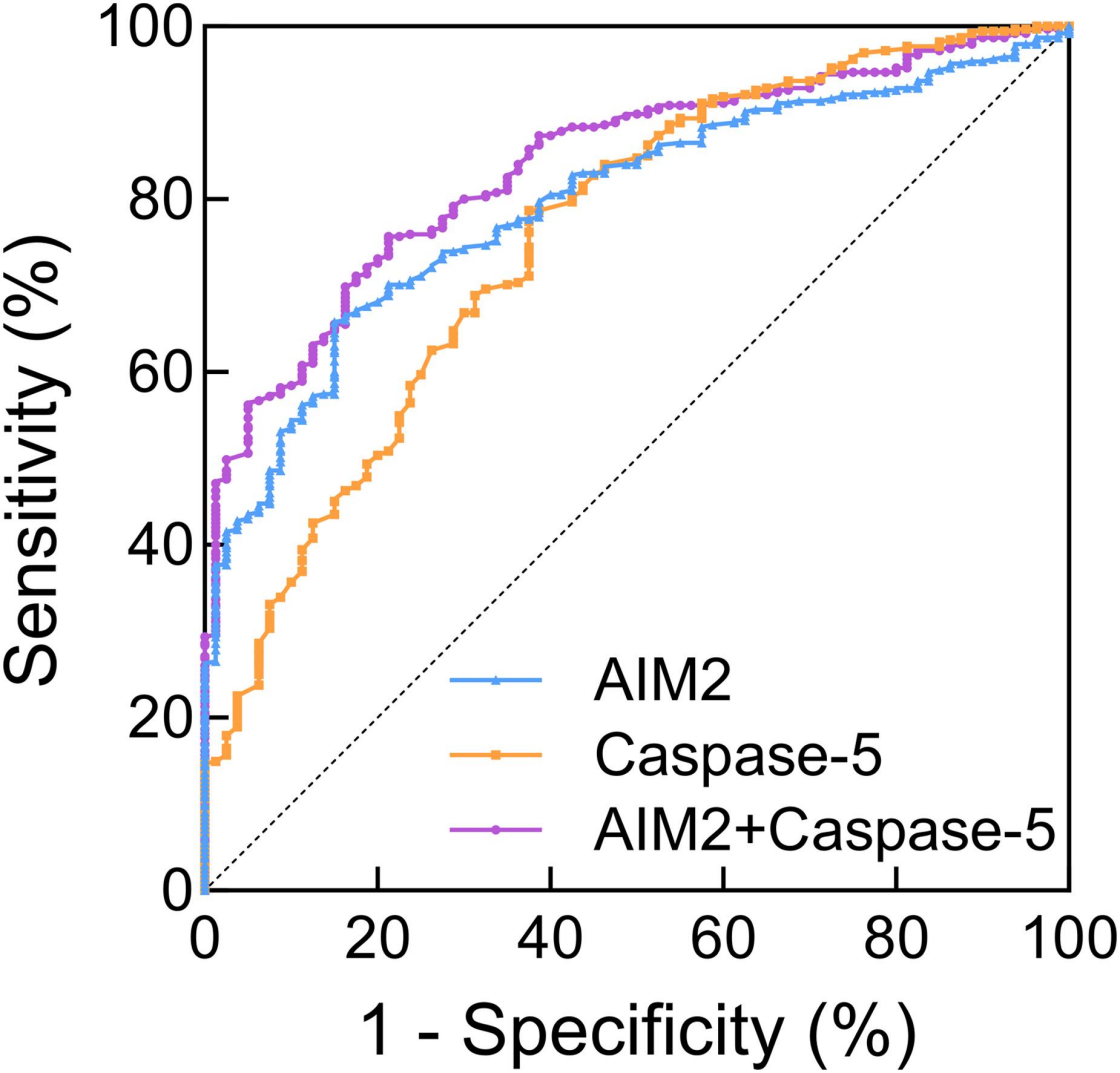
ROC curves of serum AIM2 and caspase-5 for predicting unfavorable prognosis in pediatric lobar pneumonia.

### 3.5 Assessment of incremental predictive value

To evaluate the incremental value of AIM2 and caspase-5 beyond traditional inflammatory markers, two nested logistic regression models were constructed. Model 1 (the base model) included co-infection, CRP, PCT, and LDH, with an AUC of 0.855 (95% CI: 0.817–0.893). Model 2 (the extended model) added AIM2 and caspase-5 to Model 1, with an AUC of 0.893 (95% CI: 0.863–0.923). DeLong test showed that the extended model significantly improved the AUC by 0.038 compared with the base model (Z = −1.989, *P* = 0.047), indicating that AIM2 and caspase-5 provided significant incremental predictive value over traditional markers (Figure 5).

**Figure 5:**
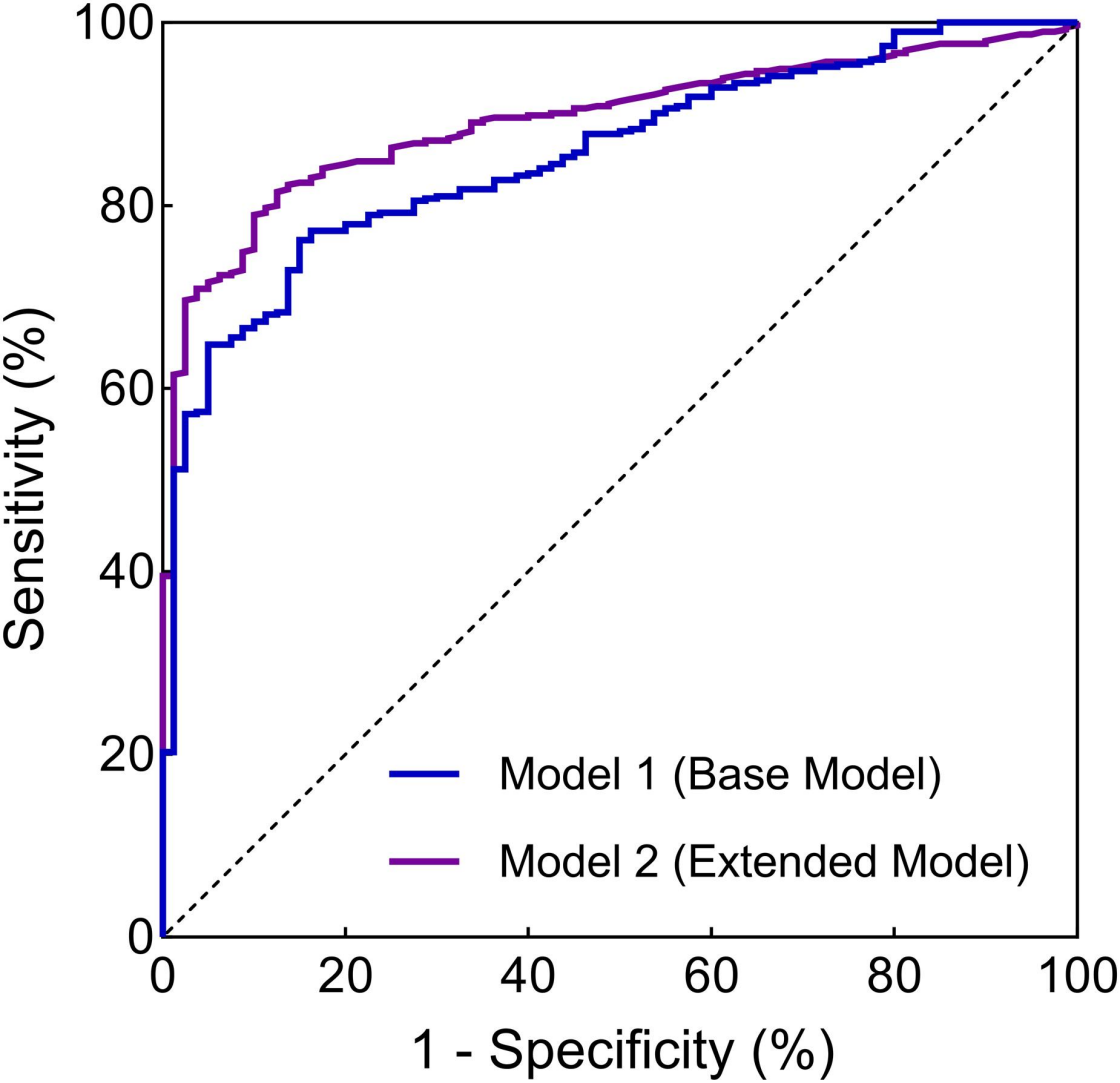
ROC curves comparing the base model and the extended model.

## 4 Discussion

Lobar pneumonia is an acute inflammatory disease characterized by lobar or segmental consolidation. Antibiotic therapy remains the cornerstone of clinical management, and most children achieve favorable prognosis (16, 17). However, some children may experience unfavorable prognosis in the setting of a complicated clinical course or delayed treatment. Therefore, the identification of reliable biomarkers for early recognition of high-risk children is of significant clinical importance.

This study analyzed the clinical and follow-up data of 475 children with lobar pneumonia and identified 80 children with unfavorable prognosis. Serum AIM2 and caspase-5 levels were significantly elevated in the unfavorable-prognosis group. The possible mechanisms are as follows. In lobar pneumonia, AIM2 functions as an important intracellular DNA sensor that recognizes cytoplasmic dsDNA and activates the inflammasome, thereby promoting IL-1β maturation and inflammatory responses and contributing to host defense against bacterial infection (9, 12). The activation mechanism of the AIM2 inflammasome has been validated in multiple disease models (18, 19), including non-infectious models such as abdominal aortic aneurysm (20) and retinal injury (21); however, its specific role in pediatric lobar pneumonia requires further lung-specific studies for confirmation. Furthermore, caspase-5 is considered an integrator of endogenous and exogenous inflammatory signals and plays a critical role in cell death and inflammation (22, 23).

Pearson correlation analysis revealed that AIM2 and caspase-5 levels were positively correlated in children with lobar pneumonia. Furthermore, logistic regression analysis demonstrated that elevated serum levels of both proteins were independently associated with an increased risk of unfavorable prognosis. Notably, AIM2 and caspase-5 are associated with the canonical (dsDNA-sensing) and noncanonical (cytosolic LPS-sensing) inflammasome activation pathways, respectively, and changes in their serum levels were consistent with the activation status of the corresponding pathways (9). However, as this study was based on single-time-point observational data, causality cannot be established, and the potential synergistic effects of other inflammasome pathways cannot be excluded. ROC curve analysis indicated that AIM2 and caspase-5 exhibited high predictive efficacy for unfavorable prognosis, further supporting their potential value in prognostic assessment of lobar pneumonia. Moreover, studies have shown that inflammasome activation plays an important role in various pulmonary infectious diseases, and the intensity of the inflammatory response it triggers is closely related to disease severity and prognosis (24, 25). As sensors and effector proteases of inflammasome pathways, AIM2 and caspase-5 are associated with the inflammasome activation process, providing broader theoretical support for the findings of this study. Notably, the combination of traditional inflammatory markers (CRP, PCT, LDH) and co-infection has demonstrated high predictive efficacy (AUC = 0.855). The addition of AIM2 and caspase-5 further increased the AUC to 0.893 (DeLong test, *P* = 0.047), demonstrating their incremental predictive value within the established marker panel. Although the improvement was modest (0.038), this enhancement remains clinically meaningful given that traditional markers already achieved high predictive efficacy, supporting the incorporation of AIM2 and caspase-5 as complementary biomarkers into clinical risk assessment rather than replacing conventional indicators.

Logistic regression analysis also revealed that co-infection and elevated levels of inflammatory markers were independent risk factors for unfavorable prognosis, suggesting that these factors provide an important reference for prognostic evaluation. For children with co-infection, comprehensive etiological work-up is recommended early after admission, with rational selection of antimicrobial agents based on identified causative microorganisms, plus combination therapy, when necessary, to reduce the risk of clinical complexity resulting from multiple infections (26, 27). Nutritional support should also be strengthened to enhance immune function and facilitate recovery. Future studies may further explore the interaction mechanisms among these risk factors and identify additional potential prognostic factors, providing stronger evidence for improving the prognosis of children with lobar pneumonia.

Furthermore, this study found that symptom duration was significantly longer in the unfavorable-prognosis group than in the favorable-prognosis group (Table 1), suggesting that such prolongation may be associated with disease severity. For children presenting with persistent high fever and progressive symptoms at admission, close monitoring of respiratory, circulatory, and other vital physiological functions is essential, with timely adjustment of treatment regimens, to prevent irreversible damage to lung tissue and other organs caused by prolonged pathological conditions (28, 29).

Despite the findings of this study, several limitations should be acknowledged. First, our sample was small, comprising a prospective cohort from only two centers, thereby restricting regional representativeness. Future studies should expand the sample size and conduct multicenter research to improve the generalizability of the results. Second, this study employed a within-case comparative design (favorable vs. unfavorable prognosis) and did not include a healthy pediatric control group; therefore, we were unable to determine whether serum AIM2/caspase-5 levels in children with lobar pneumonia are higher than healthy baseline values. Hu et al. (15) have demonstrated that serum AIM2 is elevated in children with CAP compared with healthy controls (*n* = 100); however, baseline levels in the lobar pneumonia subtype require further investigation. Third, this study utilized a single ELISA platform for detection. Although the kit manufacturer provided validation data regarding specificity and precision (intra-assay and inter-assay CVs < 10%, with no significant cross-reactivity with related proteins), the results were not verified by independent methods such as Western blotting or mass spectrometry. Future research should incorporate multi-platform validation to enhance data reliability. Moreover, the reported AUC of the ROC curve was not internally validated using bootstrap resampling, nor was it externally validated; thus, its robustness remains to be confirmed. Fourth, the study outcome was radiographic recovery at 4–6 weeks after discharge, without assessment of long-term pulmonary function, quality of life, or recurrence rates; thus, the impact on the long-term health status of these pediatric patients cannot be inferred. Fifth, as a clinical observational study, this work did not incorporate cellular experiments or animal models to further elucidate the mechanistic roles of AIM2 and caspase-5 in lobar pneumonia, optimize detection methods, or explore additional prognosis-related factors, thereby failing to provide stronger support for precision treatment and rehabilitation in children with lobar pneumonia.

In summary, our analysis of clinical characteristics and serum AIM2 and caspase-5 levels in children with lobar pneumonia demonstrates that these biomarkers are useful predictors of unfavorable prognosis, facilitating early identification of high-risk children and informing individualized management. Children with elevated biomarker levels should be considered for prompt intervention, including intensified anti-infective and immunomodulatory therapy. Furthermore, both co-infection and elevated inflammatory marker levels were identified as independent risk factors for unfavorable prognosis, underscoring the need for comprehensive treatment strategies that take these risk factors into account and integrate multidisciplinary collaboration and optimized nursing care to promote recovery.

## Data Availability

The original contributions presented in the study are included in the article/Supplementary Material, further inquiries can be directed to the corresponding author.
